# Facilitators and Barriers of the Use of Prognostic Models for Clinical Decision Making in Acute Neurologic Care: A Systematic Review

**DOI:** 10.1177/0272989X251343027

**Published:** 2025-06-29

**Authors:** Ellen X. Y. Hu, Evelien S. van Hoorn, Isabel R. A. Retel Helmrich, Susanne Muehlschlegel, Judith A. C. Rietjens, Hester F. Lingsma

**Affiliations:** Department of Health Services Management & Organisation, Erasmus University, Rotterdam, the Netherlands; Department of Health Services Management & Organisation, Erasmus University, Rotterdam, the Netherlands; Department of Clinical Genetics, Erasmus University Medical Center, Rotterdam, the Netherlands; Departments of Neurology, Anesthesiology/Critical Care Medicine and Neurosurgery, John Hopkins School of Medicine, Baltimore, MD, USA; Department of Public Health, Erasmus University Medical Center, Rotterdam, the Netherlands; Department of Industrial Design, Delft University of Technology, Delft, the Netherlands; Department of Public Health, Erasmus University Medical Center, Rotterdam, the Netherlands

**Keywords:** Prognostic models, acute neurological care, implementation, clinician perspectives, systematic review

## Abstract

**Background:**

Prognostic models are crucial for predicting patient outcomes and aiding clinical decision making. Despite their availability in acute neurologic care, their use in clinical practice is limited, with insufficient reflection on reasons for this scarce implementation.

**Purpose:**

To summarize facilitators and barriers among clinicians affecting the use of prognostic models in acute neurologic care.

**Data Sources:**

Systematic searches were conducted in Embase, Medline ALL, Web of Science Core Collection, and Cochrane Central Register of Controlled Trials from inception until February 2024.

**Study Selection:**

Eligible studies included those providing clinicians’ perspectives on the use of prognostic models in acute neurologic care.

**Data Extraction:**

Data were extracted concerning study characteristics, study aim, data collection and analysis, prognostic models, participant characteristics, facilitators, and barriers. Risk of bias was assessed using the Qualsyst tool.

**Data Synthesis:**

Findings were structured around the Unified Theory of Acceptance and Use of Technology framework. Identified facilitators included improved communication with patients and surrogate decision makers (*n* = 9), reassurance of clinical judgment (*n* = 6) perceived improved patient outcomes (*n* = 4), standardization of care (*n* = 4), resource optimization (*n* = 3), and extension of clinical knowledge (*n* = 3). Barriers included perceived misinterpretation during risk communication (*n* = 3), mistrust in data (*n* = 3), perceived reduction of clinicians’ autonomy (*n* = 3), and ethical considerations (*n* = 2). In total, 15 studies were included, with all but 1 demonstrating good methodological quality. None were excluded due to poor quality ratings.

**Limitations:**

This review identifies limitations, including study heterogeneity, exclusion of gray literature, and the scarcity of evaluations on model implementation.

**Conclusions:**

Understanding facilitators and barriers may enhance prognostic model development and implementation. Bridging the gap between development and clinical use requires improved collaboration among researchers, clinicians, patients, and surrogate decision makers.

**Highlights:**

- This is the first systematic review to summarize published facilitators and barriers affecting the use of prognostic models in acute neurologic care from the clinicians’ perspective.
- Commonly reported barriers and facilitators were consistent with several domains of the Unified Theory of Acceptance and Use of Technology model, including effort expectancy, social influence, and facilitating conditions, with the focus on the performance expectancy domain.
- Future implementation research including collaboration with researchers from different fields, clinicians, patients, and their surrogate decision makers may be highly valuable for future model development and implementation.

Neurologic disorders are one of the leading causes of disability and the second leading cause of mortality worldwide.^1^ Acute neurologic disorders refer specifically to neurologic conditions that have a sudden onset and require urgent medical attention. Examples of acute neurologic disorders include traumatic brain injury (TBI), stroke, and subarachnoid hemorrhage, which represent a significant burden on society and health care systems.^2,3^ These conditions can result in severe disability or even death if not managed promptly and effectively.

The ability to accurately predict individual patient outcomes in acute neurologic conditions is therefore of importance for clinical practice. Acute neurologic conditions are heterogeneous in terms of cause, severity, and prognosis. Consequently, the heterogeneous nature leads to challenges in the field of prognostic research since disease pathways and individual outcomes can be highly variable.^4^ Furthermore, clinical decision making in acute neurologic care can be challenging, given that patients often do not have the capacity to make medical decisions themselves, excluding them from discussions about their treatment.^5^ Therefore, family members or other surrogate decision makers are asked to assume the role of surrogate decision makers and use substituted judgment on behalf of the patient but are often too shocked or unprepared to do so. As a result, surrogate decision makers might not be able to apply substituted judgment appropriately to foresee which course of treatment the patient would prefer.^6^

In these acute situations, prognostic models may be applied to predict potential patient outcomes. A prognostic model is a combination of multiple prognostic factors that predict a specific endpoint of future clinical outcome in individual patients.^7^ The prognostic model converts the combination of predictive variables to estimate the risk of an endpoint within a specific period.^8^ The use of prognostic models may thereby support early decision making, including triage, decisions on offering high-risk procedures, inclusion, or exclusion into research studies, as it aims to provide as accurate predictions as possible to inform health care providers, patients, and their surrogate decision makers. In addition, prognostic models enhance shared decision making.^8^

Several prognostic models have been developed for a range of acute neurologic conditions,^9^ including the Corticosteroid Randomization After Significant Head Injury (CRASH)^10,11^ and the International Mission for Prognosis and Clinical Trial (IMPACT)^10^ for TBI and the National Institutes of Health Stroke Scale (NIHSS) for patients with acute ischemic stroke.^12^

While multiple well-validated and high-quality prognostic models are available to predict the clinical outcome following acute neurologic disorders,^9^ only a few models are routinely used in clinical practice.^8^ Research exploring the reasoning behind the scarce implementation of prognostic models in acute neurologic care is lacking. Reflection on the lack of implementation and use of such models is sorely needed. This systematic review aims to summarize the published facilitators and barriers perceived by clinicians that affect the use of prognostic models in clinical care for acute neurologic disorders.

## Methods

### Data Sources and Searches

This systematic review was registered in PROSPERO (CRD42022359950) and was reported according to the Preferred Reporting Items for Systematic Reviews and Meta-Analysis (PRISMA) guidelines.^13^ In consultation with 2 biomedical information specialists from Erasmus MC, we formulated the search string and conducted a systematic search across the databases Embase, Medline ALL, Web of Science Core Collection, and Cochrane Central of Register of Controlled Trials from inception until February 2024 (see Appendix 1 for the full strategies). Studies reporting facilitators and barriers regarding the use of prognostic models from clinicians’ perspective in acute neurologic care were eligible for inclusion.

### Study Selection

The search strategy prioritized the most prevalent acute neurologic conditions to capture a broad yet focused dataset within a scope that reflects the common challenges encountered in clinical practice. Studies were included based on the following inclusion criteria: 1) empirical studies in acute neurologic care settings, 2) published in English journals, 3) involvement of acute neurologic disorders (including TBI, cerebral infarction, stroke, hemorrhage, and hypoxic–ischemic brain injuries), 4) use of prognostic models or decision aids based on prognostic models (including risk prediction models, risk functions, and decision support models), and 5) provision of insight of clinicians’ perspective regarding the use of prognostic models. No restrictions regarding the age of the articles were applied. Studies were excluded when no full text was available, when publication was in a non-English language, or when the main outcome of interest of the prognostic model was not related to acute neurologic conditions. Commentaries, letters or conference abstracts, theoretical articles, and other nonempirical studies were also excluded. Reference lists of eligible articles were assessed to identify additional relevant articles.

### Data Extraction and Risk-of-Bias Assessment

Articles retrieved from the initial search strategy were imported and deduplicated in Covidence.^14^ Two independent reviewers (E.H. and E.S.v.H.) screened the retrieved articles based on predefined eligibility criteria (see Appendix 2 for the predefined eligibility criteria during the screening process). Any nonconsensus or ambiguity between the 2 reviewers was resolved with a third independent reviewer present (H.F.L.).

A tailored extraction framework was developed by the first reviewer (E.H.) in Microsoft Excel. After approval by the second reviewer (E.S.v.H.), it was used to extract data from the included articles, including 1) study characteristics, 2) study aim, 3) method of data collection and analysis, 4) applied prognostic model, 5) participant characteristics, and mentioned 6) facilitators and 7) barriers.

The Template for Intervention Description and Replication (TiDieR) checklist^15^ was applied and filled in by the first reviewer to systematically and comprehensively report the prognostic models used in the included studies. The TiDieR checklist ensures a structured and transparent approach to capturing key details about the interventions and their replicability. When the original articles lacked complete information on specific TiDieR items, related sources such as validation studies and protocols cited within the original articles were consulted. The extracted information was then incorporated into the reporting table to enhance clarity and completeness. (The completed TiDieR checklist can be found in Appendix 3.)

The risk of bias of each included article was independently assessed by E.H. and E.S.v.H. The quality of the articles was evaluated using the QualSyst tool.^16^ The Qualsyst tool consists of 2 checklists for the quality assessment of quantitative and qualitative studies. The checklist for qualitative studies consists of 10 items, whereas the checklist for quantitative studies consists of 14 items. Each item was rated on either a 3-point or dichotomous scale, with higher scores indicating lower risk of bias. The total sum of the items was divided by the maximum sum of the corresponding checklist, which depended on the methodological approach of the study (maximum sum score of 28 for quantitative studies and 20 for qualitative studies^16^). In case of a mixed-method study approach, both checklists were applied, and the total sum was divided by the maximum score of the combined checklists. Depending on the final score, the included studies were defined as having a strong (score > 0.8), good (0.71–0.80), adequate (0.51–0.70), or low (<0.5) methodological quality.^17^ Any disagreement between the first and second reviewer over the risk of bias was resolved through discussion until consensus was reached.

### Data Analysis

**Figure 1:**
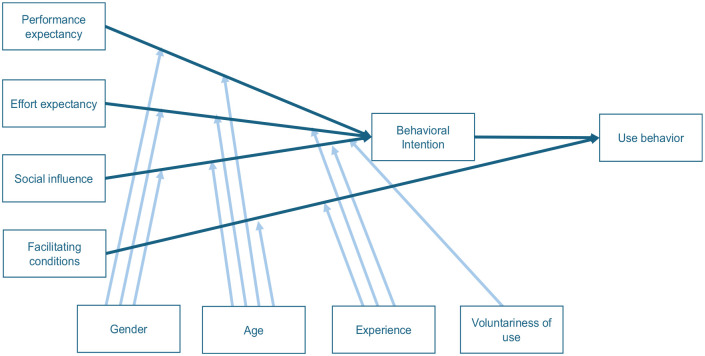
The Unified Theory of Acceptance and Use of Technology (UTAUT) framework. The perceived likelihood of adopting a technology is dependent on 4 key constructs: performance expectancy, effort expectancy, social influence, and facilitating conditions. The effect of these domains is moderated by age, gender, experience, and voluntariness of use.^18^ Source: Adapted from Venkatesh et al.^18^

Reported facilitators and barriers were structured and categorized around the Unified Theory of Acceptance and Use of Technology (UTAUT) framework (Figure 1).^18^ The UTAUT framework examines users’ acceptance and adoption of technology and suggests that usage of technology is determined by behavioral intention.^18^ The likelihood of actual usage and adoption of technology is dependent on the effect of 4 constructs: 1) performance expectancy: clinicians’ belief that prognostic models enhance patient outcomes, decision making, and workflow efficiency; 2) effort expectancy: the perceived ease of using prognostic models in fast-paced, high-pressure neurologic settings; 3) social influence: the impact of colleagues, institutional leaders, and guidelines on clinicians’ adoption of prognostic models; and 4) facilitating conditions: the extent to which clinicians perceive that organizational and technical infrastructure and resources exist to support the use of prognostic models.^18^

Derived from 8 theories and 32 constructs, the UTAUT framework has a minimum amount of complexity given the limited number of constructs and moderating variables, which makes it an applicable framework to understand acceptance behavior to any new technology.^19^

## Results

### Study Selection

**Figure 2:**
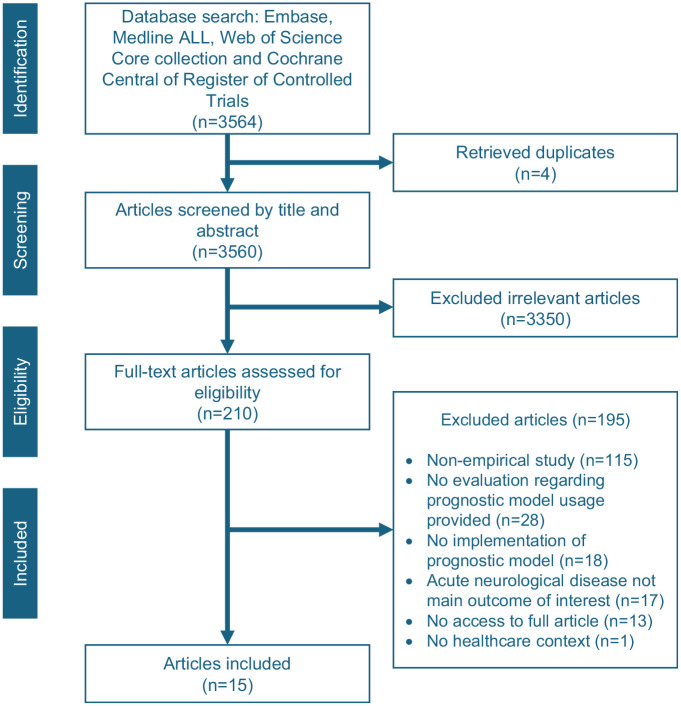
PRISMA flowchart of the literature search and selection.

A total of 3,564 studies were obtained through the systematic literature search. After screening based on title and abstract, 3,350 studies were excluded. The full text of the remaining studies was retrieved for further assessment, of which 195 studies were excluded due to the following reasons: not being an empirical study (*n* = 115), no qualitative evaluation about the prognostic model provided (*n* = 28), no implementation of the prognostic model mentioned (*n* = 18), TBI or other acute neurologic disease was not the main outcome (*n* = 17), no access to the full article (*n* = 13), non-English publication (*n* = 3), and no health care–related context (*n* = 1). An updated literature search for additional articles in February 2024 identified 4 potential studies that were considered eligible. In total, 15 articles were included for data extraction and analysis (Figure 2). These 15 articles covered 11 prognostic models, with further details provided in the TiDieR checklist (Appendix 3).

### Study Characteristics and Methodological Quality

All included studies were published between 2013 and 2024, including qualitative (*n* = 6),^20–25^ quantitative (*n* = 3),^26–28^ and mixed-methods studies (*n* = 6)^29–34^ (Table 1). Studies were performed in North America (*n* = 11),^21–24,26,28,29,31–33^ Europe (*n* = 1),^20^ Oceania (*n* = 1),^27^ Asia (*n* = 1),^34^ Africa (*n* = 2),^30^ and North America and Africa (*n* = 1).^25^ Clinical settings of the articles included nonacademic hospitals (*n* = 7),^20,25,26,28,30,31,33^ academic hospitals (*n* = 4),^21,23,29,34^ both academic and nonacademic hospitals (*n* = 2),^22,24^ general practice (*n* = 1),^27^ and health care in general (*n* = 1).^32^ Five of the 15 studies evaluated the implementation of a model^26,27,30,31,34^; the other studies explored the perceived facilitators and barriers regarding the usage of prognostic models. Evaluated models included clinical decision support (*n* = 9),^20–22,26–29,33,34^ prognostic models (*n* = 3),^23,25,30^ decision aids (*n* = 2),^31,32^ and prediction rules (*n* = 1).^24^ Participants were mostly physicians^20–24,26,28–31,33^ and nurses.^24–26,28,29,31^ The methodological quality of the studies was scored as strong (*n* = 9),^20–24,28,29,32^ good (*n* = 3),^26,30,34^ adequate (*n* = 2),^31,33^ and low (*n* = 1)^27^ (Appendix 4). No studies were excluded based on their quality rating. The study with a low-quality rating was included as it provided relevant new insights.

**Table 1:** Characteristics of the Included Studies

First Author, Year of Publication, Region	Study Aim	(A) Method of Data Collection (B) Method of Data Analysis	Clinical Setting	(A) Condition Types (B) Patient Population	Prognostic Model (Type, Form, and Provided Outcome)	Participant Characteristics (*n*; Professions (Physician and Other Clinical Occupations); Gender; Mean Age (Range or SD)	Mentioned Facilitators	Mentioned Barriers
Amann, 2023, Germany and Switzerland^20^	To complement existing normative guidelines and ethical frameworks by empirically probing stakeholders’ views on ethically relevant issues relating to the use of medical AI in a concrete clinical context, namely, stroke medicine	(A) Semi-structured interviews, exploring perceptions toward AI-powered CDSS for stroke (B) Deductive thematic analysis	Hospital	(A) Stroke (B) Adults	AI-based CDSS, providing diagnosis assistance, treatment recommendations, and outcome predictions^a^	*N* = 14, includingphysicians (*n* = 7), occupational therapist (*n* = 1), psychotherapist (*n* = 4), neuropsychologist (*n* = 2) 64% female Age 62 y (47–80 y)	(1) Enhancement interprofessional and patient communication (2) Provision of structure, faster and accurate diagnosis	(1) Need for access to relevant patient information (2) Dehumanization of health care (3) Challenging clinicians’ authority (4) Unclear responsibility (5) Medical error due to errors in data
Elahi, 2020, Uganda^30^	To evaluate the feasibility and acceptability of implementing an SSA-based TBI risk calculator at 2 referral hospitals in Uganda	(A) Surveys and semi-structured interviews, evaluating the implementation and usage of the risk calculator (B) Descriptive statistics and inductive content analysis	Hospital	(A) TBI (B) NA	SSA-based TBI decision support system, presented as a mobile and Web-based application, providing risk scores for poor outcomes and decision support for clinical decisions	*N* = 28, including: physicians (EM *n* = 5, intern doctor *n* = 11), general surgery (*n* = 6), neurosurgery (*n* = 6) 24% female Age 30.3 y (SD 6.7 y)	(1) Simplicity of interface (2) Data points reminders (3) Provision of objective assessment in resource-limited settings	(1) Challenge to complete downstream interventions after outcome prognosis (2) Dependency on external technical factors (3) Reluctance in usage with end-of-life decisions
Flynn, 2015, UK^31^	To develop a decision aid to support patient-specific clinical decision making about thrombolysis for acute ischemic stroke and clinical communication of personalized information on benefits/risks of thrombolysis by clinicians to patients/relatives	(A) Mixed methods, including surveys and interviews, evaluating implementation and usage of the computerized decision aid (B) Conceptual content analysis	Hospital	(A) (Ischemic) stroke (B) Adults	Decision support system, presented as a software-based clinical tool, providing risk probabilities for stroke outcomes and treatment impact predictions for personalized clinical decision making	*(Usability testing)* *n* = 27, including: physicians (stroke physicians *n* = 5, emergency department physicians *n* = 2), stroke nurses (*n* = 5), other (patients *n* = 8, relatives *n* = 7) *(Feasibility testing)* *n* = 19, including: stroke physicians and stroke nurse practitioners^b^	(1) Efficacious mode of decision support (2) Enhancement in risk communication with intuitive visual presentations	(1) Clinicians’ acceptance of outcome probabilities (2) Capability of patient/relatives to understand risk presentations (3) Interruption of clinical workflow
Ghandour, 2020, Canada^32^	To create consensus among Canadian mild traumatic brain injury and EM experts on medications required to adapt 2 American decision aids about head CT use for adult and pediatric mild traumatic brain injury to the Canadian context	(A) Nominal group technique consensus and focus groups, exploring perceptions on needed adaptations for the decision aids (B) Inductive qualitative analysis	General health care setting	(A) (Mild) TBI (B) First model: adults, second model: pediatric	Decision aid, presented as paper guide and digitally, providing information and guidance to support patient decision making	*N* = 21, including: physicians (EM *n* = 5), other (researchers *n* = 8, decision maker *n* = 1, patients’ representatives *n* = 3, students *n* = 4)^b^	(1) When decision aids can be used to reduce litigation risk (2) Reduction in CT scan use	(1) Risk of increased anxiety in patients when presenting findings
Greenberg, 2021, US^21^	(1) To investigate the sociotechnical influences on implementing electronic CDS to guide clinicians’ decisions regarding the need for ICU admission (2) To investigate alternative uses of electronic CDS among children with MHT and ICI	(A) Semi-structured focus groups, exploring perceptions on development and implementation of CDS (B) Inductive thematic analysis	Academic hospital	(A) MHT and ICI (B) Pediatric	Clinical decision support system, presented and integrated as a software tool, providing management of children with TBI and intracranial injuries	*N* = 32, including: physicians (EM *n* = 9), neurosurgery (*n* = 11), general surgery (*n* = 3), critical care (*n* = 3), informational technology (*n* = 4), other (*n* = 2) 38% female, no answer (*n* = 1) Age 30–39 y (*n* = 17), age 40–49 y (*n* = 10), age 50–59 y (*n* = 4), no answer (*n* = 1)	(1) Standardization and supporting consensus decisions across services (2) Support of family counseling (3) Expanding risk knowledge	(1) Conflicts in following outcome due to limited resources (2) Interdepartment and workflow conflicts (3) Unclear repercussions when outcome are not followed (4) End-users missing expertise to use prognostic tool
He, 2023, China^34^	(1) To provide a set of systematic design guidelines by identifying, compiling, and sorting design suggestions in the existing AI-CDSS literature (2) To apply the AI-CDSS design guidelines to guide the design practice and produce an AI-CDSS for stroke thrombolysis risk assessment	(A) Systematic review and mixed methods (semi-structured interviews and questionnaires) to evaluate the design of the AI-CDSS among end-users (B) Narrative analysis, thematic analysis and descriptive statistics	Academic hospital	(A) Stroke (B) Adults	AI-based CDSS, presented as a software-based system, providing thrombolysis risk assessment and therapy advice	*N* = 6, including: associate chief physician (*n* = 1) Physician in charge (*n* = 4) House physician (*n* = 1) 33% female^2^	(1) Alignment with current clinical workflow, facilitating integration (2) Improvement of patient communication through intuitive risk presentation (3) Usage of the tool leads to extra confidence in clinical decision making	(1) Increase in workload due to data input
Kiatchai 2017, USA^26^	To develop, implement, and conduct feasibility evaluation of a CDS system to provide real-time decision support and guidance to anesthesiologists managing neurosurgery for pediatric TBI patients	(A) Surveys evaluating the feasibility and acceptability of implementing a CDS system (B) Descriptive cross-sectional	Hospital	(A) TBI (B) Pediatric	Real-time clinical decision support system, presented and integrated as a software-based system, providing real-time decision support and performance metrics	*N* = 44, including anesthesiology providers, including attending anesthesiologists, residents, and nurse anesthetics^b^	(1) Improved patient outcomes and communication	(No barriers found)
Liberman, 2022, USA^22^	(1) To explore EM physicians’ knowledge, attitudes, and beliefs regarding acute diagnostic neurology (2) To understand physicians’ thoughts about the use of CDS models to provide clinicians with clinical knowledge and patient-related information presented at appropriate times to enhance patient care, to improve neurologic diagnosis in the ED	(A) Semi-structured interviews, exploring perceptions about the use of CDS to improve diagnostic accuracy (B) Direct content analysis	Hospital and academic hospital	(A) Neurologic conditions in general (B) NA	Clinical decision support to aid diagnostic evaluation of patients in the ED^a^	*N* = 16 Emergency medicine physicians 38% female^b^	(1) When the tool is defensible in court (2) Easy implementation in current workflow	(1) Unwillingness to have clinical judgment affected by CDS (2) Diversion in opinions regarding usage of CDS in ED and usefulness of the tool
Masterson Creber, 2018, USA^29^	To summarize the quantitative efficacy findings of the multicenter PECARN implementation trial and integrate with additional contextual/qualitative data to identify and understand the reach, adoption, implementation, and maintenance of the EHR CT CDS tool	(A) Semi-structured interviews, summary of quantitative data sources, postimplementation semi-structured interviews, exploring perceptions and evaluating the implementation of prediction rules (B) Content analysis	Academic hospital	(A) TBI (B) First model: pediatrics (<2 y), second model: pediatrics (2–18 y)	Clinical decision support systems, presented as software-based system, providing risk estimates and recommendations for clinical decision making	*N* = 37, including: physicians (*n* = 21), staff nurses (*n* = 8), nurse practitioners/physician assistants (*n* = 2), residents (*n* = 2), other stakeholders (*n* = 5) 66% female^b^	(1) Availability of clinical champion (2) Strong perception of relevance (3) Tailored integration in site’s workflow (4) Education for informal surrogate decision maker and patient’s families	(1) Unclarity about the end user of the tool (2) Unclear usability in high-severity trauma settings
Moskowitz, 2018, USA^23^	To examine physicians’ awareness, perceptions, and use of the IMPACT model, as well as their preferences for communicating prognostic estimates during family meetings with ciTBI surrogates	(A) Semi-structured interviews, exploring perceptions on usage of the IMPACT prognostic model (B) Qualitative content analysis	Academic hospital	(A) TBI (B) Adults	Prognostic model, providing standardized outcome metrics including numeric risks^a^	*N* = 20, including: physicians (neurocritical care *n* = 10, trauma surgery *n* = 2), neurosurgery (*n* = 7), palliative care (*n* = 1) 35% female Age 47 y (SD 8 y)	(1) Reduction of physician variability in heterogeneous disease	(1) Perception that prognostic models are research tools (2) Mistrust in underlying data (3) Presenting outcome might mislead patient and informal surrogate decision maker
O’Leary, 2023 US/Tanzania^25^	To assess the feasibility and practicality of a TBI prognostic model implementation at KCMC (Tanzania) and amongst Duke (USA) affiliated health care providers using human-centered design techniques to transfer knowledge between both settings to improve the future implementation of the tool at KCMC	(A) Semi-structured interviews, exploring perceptions of the feasibility of a TBI prognostic model	Hospital	(A) TBI (B) NA	Prognostic model, presented as Web-based application, providing predicted mortality to assist treatment decisions and resource allocation	*N* = 21, including: physicians (doctors *n* = 8), residents (*n* = 3), nurses (*n* = 10) 48% female Age 34 y (8.5 y)^b^	(1) Alignment with clinical workflow (2) Training in tool usage (3) Transparency in how the model operates (4) Objective assessment in resource allocation (5) Improvement in patient communication (6) Innovative climate in hospital	(1) Concerns regarding availability of resources (2) Inequality in access to the tool (3) Increase in workload due to data input (4) Outcomes of the model does not fit with the immediate professional scope
Ranta, 2013, New Zealand^27^	To assess the feasibility of implementing the TIA/stroke EDS in the MidCentral DHB primary care sector prior to a district-wide launch	(A) Surveys evaluating the feasibility and implementation of the EDS (B) Descriptive statistics	General practice	(A) TIA (B) Adults	Electronic decision support tool^a^	*N* = 11 General practitioners^b^	(1) Usage of tool leads to extra credence to assessment	(1) Impinging on autonomy when no override options were allowed
Sheehan, 2013, USA^24^	To describe the sociotechnical environment in the ED to inform the design of a CDSS intervention to implement the PECARN clinical prediction rules for children with minor blunt head trauma	(A) Focus groups and interviews, exploring perceptions on the implementation of CDSS into electronic health records (B) Thematic analysis	Hospital and academic hospital	(A) TBI (B) Pediatrics	Clinical decision support system, presented and integrated as a software-based system, providing risk assessment and guidance support	*N* = 126, including: physicians (*n* = 42), MD residents (*n* = 8), nurses (*n* = 48), nurse managers (*n* = 13), other clinicians (*n* = 6), and IT liaisons/staff/leaders (*n* = 9)^b^	(1) Standardization of clinical care (2) Smooth integration in technical layout of ED (3) Positive interprofessional relationships (4) In line with regulatory requirements	(1) Computers not placed in a efficient way in the ED for use (2) Unclear task coordination
Yadav, 2015, USA^33^	To design a pediatric TBI eCDS tool for trauma resuscitation using a human factors approach	(A) Semi-structured interviews and surveys, exploring perceptions on the implementation of the CDS (B) Content analysis	Hospital	(A) TBI (B) Pediatrics	Clinical decision support tool, presented and integrated as software-based system, providing support in clinical decisions	*N* = 26, including: physicians (emergency physicians, pediatric EM members), clinical experts, human factor engineers^b^	(No facilitators found)	(1) Not matching the clinical workflow
Zakhari, 2016, USA^28^	(1) To assess the attitudes of clinicians toward an evidence-based CDS tool CCHR (2) To assess if the attitude of the clinician affects the likelihood of adopting a new practice guideline intended to decrease the rate of CT scans ordered	(A) Surveys exploring perceptions on the use of the CDS (B) Descriptive statistics	Hospital	(A) TBI (B) Mixed (ages 16–64 y)	Clinical decision support tool, presenting decision-making guidance^a^	*N* = 100, including: physicians (attending physicians *n* = 11), nurse practitioners (*n* = 25), physician assistants (*n* = 7), postgraduates (*n* = 8), and nurses (*n* = 49)^b^	(1) Encouragement from higher hierarchy (2) User satisfaction among colleagues	(1) Fear of being held liable when clinically important findings are missed out

AI, artificial intelligence; CCHR, Canadian CT Head Rule; CDS, clinical decision support; CDSS, clinical decision support system; ciTBI, critically ill traumatic brain injury; CT, computed tomography; DHB, district health boards; eCDS, electronic clinical decision support; ED, emergency department; EDS, electronic decision support; EHR, electronic health record; EM, emergency medicine; ICI, intracranial injuries; ICU, intensive care unit; IMPACT, International Mission For Prognosis And Clinical Trial; IT, information technology; KCMC, Kilimanjaro Christian Medical Center; MHT, minor head trauma; NA, not available; PERCARN, the Pediatric Emergency Care Applied Research Network; SD, standard deviation; SSA, Sub-Saharan Africa; TBI, traumatic brain injury; TIA, transient ischemic attack.

a Further detailed information about the prognostic model was not reported.

b Further detailed characteristics of participants were not reported.

### Identified Facilitators and Barriers

Frequently identified facilitators and barriers across the included studies were concentrated in the *performance expectancy* construct of the UTAUT framework (Table 2). The most reported facilitators and barriers are further elaborated on in the following sections.

**Table 2:** Summary of Found Facilitators and Barriers under Unified Theory of Acceptance and Use of Technology Framework Constructs

Facilitators for Implementation	Studies	Barriers to Implementation	Studies
Performance expectancy^a^			
Improved communication with patients and informal surrogate decision maker	20, 21, 24–26, 29–31, 34	Risk communication	20, 23, 31
Reassurance of clinical judgment	20, 21, 25, 27, 29, 34	Mistrust in data	20, 23, 31
Perceived improved inpatient outcomes	20, 21, 26, 30	Perceived reduction in autonomy	20, 25, 27
Standardization of care	20, 21, 23, 24	Ethical considerations	23, 30
Resource optimization	25, 30, 32		
Extension on clinical knowledge	20, 21, 34		
Effort expectancy^b^			
Simple interface	30, 32	Difficulty of use	21, 29, 30, 32
		Technical factors	24, 25, 30, 31
Social influence^c^			
Innovative work climate	25, 28, 29	Accountability	20, 21, 24
		Interprofessional conflicts	21, 24, 29
Facilitating conditions^d^			
Fitting workflow	24, 25, 29, 34	Workflow conflicts	21, 22, 24, 25, 30, 31, 33, 34
Legal context	24, 32		

a Clinicians’ belief that prognostic models enhance patient outcomes, decision making, and workflow efficiency.

b The perceived ease of using prognostic models in fast-paced, high-pressure neurologic settings.

c The impact of colleagues, institutional leaders, and guidelines on clinicians’ adoption of prognostic models.

d The extent to which clinicians perceive that organizational and technical infrastructure and resources exists to support the use of prognostic models.

### Performance Expectancy

The construct *performance expectancy* of the UTAUT framework captures clinicians’ belief that prognostic models enhance patient outcomes, decision making, and workflow efficiency. Six facilitators were identified, including improved communication with patients and surrogate decision makers (*n* = 9), reassurance of clinical judgment (*n* = 6), improved patient outcomes (*n* = 4), standardization of care (*n* = 4), resource optimization (*n* = 3), and extension on clinical knowledge (*n* = 3). Four barriers were identified, including perceived misinterpretation during risk communication (*n* = 3), mistrust in data (*n* = 3), perceived reduction of autonomy (*n* = 3), and ethical considerations (*n* = 2).

#### Facilitators

##### Improved communication with patients and surrogate decision makers

Prognostic models aided communication between clinicians, patients, and surrogate decision makers by conveying information in commonly understood language. Using prognostic models as communication framework helped clinicians feel more confident during counseling sessions.^20,21,24–26,29–31,34^ This was especially effective when family–clinician discussions were supported by verbal explanations from trusted clinicians, combined with supporting pictographs, percentages, and natural frequencies for a clear and intuitive presentation.^31^

##### Reassurance of clinical judgment

Prognostic models provided reassurance for clinicians’ clinical judgment and offered a sense of “grounding” for clinicians with less experience by supporting their decisions with more robust data.^20^ For example, clinicians reported feeling more confident when discharging patients.^21,29^ In addition, clinicians mentioned that using prognostic models added extra credibility to their assessment.^27,34^

##### Perceived improved patient outcomes

Prognostic models assisted clinicians in making objective and accurate predictions about the clinical course and outcome of patients, which informed tailored treatment decisions and potentially led to better perceived patient outcomes.^20,21,26^ One study showed that prognostic models facilitated triage decisions, specifically in settings in which the clinician performing the initial assessment was unable to rely on previous experiences or data.^30^

#### Barriers

##### Perceived misinterpretation during risk communication

The perception that prognostic outcomes might lead to misinterpretation, either through optimistic bias or by causing negative emotions among patients and surrogate decision makers, made clinicians reluctant to present prognostic outcomes during consultations.^20^ Clinicians feared that patients with low health literacy and numeracy might not be able to interpret the medical terms and statistics used in the prognostic model.^23,32^ Combined with feelings of stress and despair, presenting prognostic outcome probabilities could have led to false hope, unfounded optimism, or anxiety among patients and their surrogate decision makers.^23,31,32^

##### Mistrust in data

Clinicians raised concerns about the data quality of prognostic models. Due to the heterogeneous nature of acute neurologic conditions, clinicians doubted the data underlying prognostic models as well as the quality of the generated output.^20,23^ This included reservations about the limited selection of variables from the source studies affecting model development and the recognition of the limitation of standardized data collection across research studies.^23^ In addition, clinicians expressed difficulties accepting model outcomes for patients at the extreme values of prognostic variables.^31^ One study showed that clinicians perceived outcome scores as research tools intended to inform clinical trial design rather than tools for bedside implementation at the individual level.^23^ These concerns reflected skepticism about the quality and generalizability of the data used to develop prognostic models.

##### Perceived reduction in clinicians’ autonomy

Clinicians expressed that clinical decision-making power should remain with them (and patients), arguing that prognostic models should therefore not interfere with their epistemic authority.^20^ The introduction of these models could raise concerns about the questioning of the clinicians’ knowledge and expertise, potentially leading to perceived devaluation of professional judgment.^25^ In addition, clinicians requested the ability to override the prognostic outcome when it appeared inappropriate or when they felt the model as imping on their clinical autonomy.^20,27^

### Effort Expectancy

The construct *effort expectancy* of the UTAUT framework depicts the perceived ease of using prognostic models in fast-paced, high-pressure neurologic settings. A simple interface (*n* = 2) was identified as a facilitator. Two barriers were identified, namely, difficulty of use (*n* = 4) and technical factors (*n* = 4).

#### Facilitators

##### Simple interface

Given the hyperacute clinical setting of acute neurologic conditions, clinicians indicated that computerized technologies were likely the most effective form of delivering rapid decision support.^30^ The use of simple language, simplified descriptions, and quantification of risk outcomes was highlighted to facilitate usage.^32^

#### Barriers

##### Difficulty of use

Participants experienced difficulties interpreting and applying the prognostic output into clinical settings.^29,32^ Some clinicians mentioned that intended end users of the model lacked the expertise to correctly assign each input variable in the prognostic model.^21^ Other studies revealed that prognostic models were often misunderstood; some clinicians mistook them for electronic medical records or confused them with other unrelated tools and systems.^30,32^

##### Technical factors

Access to prognostic models was not always guaranteed in clinical settings. For example, electronic devices were not always placed in an accessible location during patient encounters.^24,30^ In resource-limited settings, the use of prognostic models was not always feasible due to dependence on a stable internet connection or the availability of technical equipment.^25,30^ Besides these external considerations, some clinicians were inexperienced with computer use in general.^31^

### Social Influence

The construct *social influence* of the UTAUT framework depicts the impact of colleagues, institutional leaders, and guidelines on clinicians’ adoption of prognostic models. An innovative work climate (*n* = 3) was identified as a facilitator. Two barriers were identified: accountability (*n* = 3) and interprofessional conflicts (*n* = 3).

#### Facilitators

##### Innovative work climate

The use of prognostic models was more likely when clinicians felt encouraged and inspired by their environment. This could be in the form of expectations from higher hierarchical levels in the workplace, including employees, supervisors, or at the governmental authorities.^25,28,29^ At the employee level, adoption of the model was more likely when close colleagues were satisfied with its use^25,28^ or when the use of the model was mandated.^25^ The implementation of clinical champions was seen as essential, as their role was to provide education and support for the use of new technologies among intended end users.^29^ Generating extrinsic motivation to enforce the use of prognostic models was proposed through the application of a benchmark report,^29^ which compared the use of the models among providers.

#### Barriers

##### Accountability

Clinicians expressed reluctance toward the use of prognostic models due to the fear of being held liable when missing clinically important findings.^20,24^ Consequently, when clinical mistakes were made, it was unclear who held responsibility and what possible (legal) consequences it could lead to if the provided outcome of the prognostic model was not followed up on.^20,21^

##### Interprofessional conflicts

The use of prognostic models in clinical practice led to conflicts between specialties, including limited added relevance for nonneurosurgeons as they sought assistance from surgeons regardless of the prognostic outcome of the model.^21^ Another barrier related to interprofessional relationships arose when decisions were made hierarchically and the prognostic model was not accessible in a shared medium, leading to conflicts in task coordination.^24,29^

### Facilitating Conditions

The construct *facilitating conditions* of the UTAUT framework depicts the extent to which clinicians perceive that organizational and technical infrastructure and resources exists to support the use of prognostic models. Two facilitators were identified, namely, fitting workflow (*n* = 4) and legal context (*n* = 2). Workflow conflicts (*n* = 8) was identified as a barrier.

#### Facilitators

##### Fitting workflow

Seamless integration into the existing clinical workflow facilitated the uptake of prognostic models in clinical practice. Automating prognostic outcomes into clinical instructions was suggested to reinforce the use of the model.^25,29,34^ In addition, the layout of the prognostic model aided integration when computers were easily accessible, for example, in shared workspaces where clinicians could build on previously collected patient information.^24^

##### Legal context

Prognostic models were more readily supported by clinicians and organizations when they complied with regulatory requirements.^24^ In addition, clinicians were also more likely to use prognostic models when they led to a reduction of litigation risk.^32^

#### Barriers

##### Workflow conflict

Implementation of prognostic models might lead to conflicts in the existing clinical workflow.^24,31,33^ Challenges arose in terms of completing follow-up interventions after the prognostic outcome, particularly when resources were limited given the suggested outcome of the model.^21,30^ Consequently, clinicians perceived the additional task of managing patient and surrogate decision makers’ expectations as burdensome when the suggested treatment could not be provided.^30^ Furthermore, the outcome of the model should align with the scope of interest of the end user.^22,25^ One study mentioned that in emergency settings, clinicians primarily aimed to understand the patient’s situation to determine the next course of action rather than obtaining a precise prognosis. Based on that theme, prognostic models that provide diagnostic accuracy rather than guiding actionable next steps were less likely to be adopted by clinicians.^22^

## Discussion

In this systematic review, we summarized the facilitators and barriers for the use of prognostic models in the acute clinical care of neurologic disorders from the clinicians’ perspectives. Through a systematic search, we identified various factors that might influence the use of prognostic models. We synthesized and categorized the findings of this study using the UTAUT framework.^18^ Overall, commonly mentioned facilitators included 1) improvements in communication with patients and surrogate decision makers, 2) reassurance of clinical judgment, 3) perceived improvement in patient outcomes, 4) the simple interface of the prognostic model, 5) innovative work climate, and 6) fit into current workflows. Barriers included 1) perceived misinterpretation during risk communication with patients and surrogate decision makers, 2) mistrust in the data of the prognostic model, 3) perceived reduction of autonomy, 4) perceived difficulty of use, 5) technical factors, 6) accountability, 7) interprofessional conflicts, and 8) workflow conflicts.

In line with previous studies, our study suggests that prognostic models aid risk communication with patients and their surrogate decision makers^35^ when presenting a prognostic outcome in an intuitive and understandable manner.^36,37^ Prior studies suggest ways to facilitate risk communication, including presenting absolute risk frequencies instead of relative risk and incorporating simple language and pictographs.^38^ Earlier studies have suggested that the unique dynamics of pediatric and adult care may necessitate different approaches when using prognostic models.^39,40^ While the studies in this review did not find clear evidence of significant differences in facilitators and barriers between these groups, factors such as caregiver involvement in pediatric care and clinician–patient communication in adult care may influence the effectiveness and perception of these tools.

Consistent with prior literature, clinicians were reluctant to discuss prognostic outcomes during clinician–family counseling due to fears of causing distress, optimistic bias among patients and surrogate decision makers,^41^ or concerns about the perceived low literacy and numeracy within these populations,^38,42^ ultimately leading to fears of misinterpretation or unfounded optimism in prognostic outcomes. These findings highlight the importance of tailoring risk communication to the health literacy levels of patients and surrogate decision makers.^43,44^ In addition, clinicians must discuss the inherent uncertainties in clinical prediction models, ensuring that patients and surrogate decision makers understand the limitations and applicability of prognostic information.^43^

Furthermore, this systematic review revealed that limited trust and knowledge in prognostic models pose major barriers to implementation. Given the complexity of these models, which incorporate multiple variables and statistical algorithms, clinicians may struggle to use, interpret, and understand their output. Consistent with previous studies, clinicians were also more likely to adopt prognostic models when they understood these models as enhancing rather than replacing their clinical judgment.^35,45^ Beyond these practical barriers, the ethical implications of prognostic models require balancing patient autonomy and clinician judgment. These tools should complement,^46^ not replace, clinical decision making while respecting patient values and preferences.^46^ Overreliance on models may overlook the nuances of care, making an ethical framework emphasizing shared decision making, informed consent, and patient empowerment essential for responsible use and maintaining the clinician–patient relationship.^44^

User-friendly and clinically comprehensible indicators could simplify prognostic model use.^47^ Providing clinicians with technical and procedural support, including an approachable clinical champion on site, can help bridge knowledge gaps and shift attitudes toward the use of these models.^48^ Our study also highlights the importance of a seamless integration into clinicians’ current workflows. Clinicians prefer models that help determine next steps rather than those focused solely on diagnosis. This aligns with prior research emphasizing the need to integrate model outputs into electronic health records,^49^ clinical pathways, and downstream interventions for effective implementation.^45,49^ External factors such as emergency department layout, reliance on technical devices, and resource limitation should be addressed to minimize disruptions in clinicians’ workflow and minimize additional burden in clinicians’ workload. Beyond practical integration, legal and accountability concerns are critical. Clinicians may hesitate to rely on models lacking transparency or clinical credibility due to fears of liability in cases of subclinical decision making or clinical mistreatment.^50^ Clearer guidelines on the legal and clinical implications of prognostic model use are therefore needed.

### Strengths and Limitations

This is the first systematic review that explored published facilitators and barriers among clinicians regarding the use of prognostic models for clinical decision making in acute neurologic care. The strengths of this review include its methodological approach, using the UAUT framework as an analytical model for further examination, and categorization of the found facilitators and barriers. In addition, most of the included articles were assessed as high quality, enhancing the credibility of the findings.

However, when interpreting the outcomes of this review, potential limitations should be considered. One key limitation is the inherent heterogeneity of the included studies, including differences in geographic locations, hospital settings, resource access, patient demographics, and prognostic models. Given this variability and the inclusion of 15 articles on 11 prognostic models, meaningful subgroup analysis was not feasible. Factors such as high- versus low-income countries,^51^ resource constraints,^51^ and clinician experience^52^ may influence perceptions and use of prognostic models. In addition, cultural factors, including attitudes toward patient autonomy in shared decision making, further shape adoption and perceived utility.^53^ The inclusion of various studies incorporating different prognostic models contributes to the heterogeneity of this systematic review, necessitating cautious interpretation when applying findings across different healthcare contexts. While some common trends can be identified, the limited number of studies for each model type makes it difficult to fully assess differences in barriers and facilitators between them, thus preventing a detailed comparative analysis. Trends suggest that artificial intelligence–based CDSS and CDSS share challenges related to data trust and clinician autonomy, while decision aids and prognostic models face greater ethical concerns about patient involvement.

Another limitation is the small sample size and variability in participant roles within each included study. In many studies, the number of clinicians involved was not specified, which may affect the generalizability of our findings.

Moreover, the focus on acute neurologic care can be considered another limitation. However, many identified facilitators and barriers, such as trust in data, communication challenges, and workflow integration, may also apply to other settings such as intensive care and emergency medicine. These fields similarly involve high-stakes, time-sensitive decision making and complex prognostic models. This indicates the broader relevance of our findings while recognizing unique prognostic uncertainties, including high patient variability, delayed recovery, and the influence of treatment decisions on outcomes.^54,55^ In addition, although this review primarily focuses on common acute neurologic conditions, including rarer conditions could reveal unique barriers, such as limited data and access to specialized tools. However, many of the identified challenges in this review, such as risk communication, clinical judgment, workflow integration, and trust in data, are shared across both common and rare neurologic conditions. We therefore believe that the identified barriers and facilitators are likely to be applicable to rarer conditions as well.

A further limitation is the scarcity of studies evaluating the actual implementation and usage of prognostic models. Only 5 of the 15 articles provided insights into clinicians’ actual usage of prognostic models, while the remaining articles focused on perceived facilitators and barriers. While clinicians’ perceptions provide valuable insights, their subjective nature may limit the applicability of the findings across different clinical settings. This highlights the importance of future studies incorporating objective measures such as implementation rates and adherence to clinical guidelines alongside qualitative insights to ensure a more robust understanding of prognostic model usage in acute clinical care.^56^

Finally, while we excluded gray literature to maintain methodological rigor, we acknowledge that this may limit the comprehensiveness of our review, particularly regarding real-world implementation challenges. Although the gray literature often lacks standardized reporting and quality assessments, it can provide valuable insights into practical barriers clinicians face, which peer-reviewed studies may overlook.

### Future Research Directions

The number of published implementation studies regarding prognostic models in acute neurologic care remains small, despite the extensive development and external validation of such models. Most existing literature focuses on model development rather than practical application, leaving a critical gap in understanding their practical utility. Given that most studies in this review were conducted in high-income countries, future research should validate these findings in more diverse health care contexts. In addition, expanding research to less common conditions could provide a more comprehensive understanding of the use of prognostic models across a wider range of acute neurologic disorders.

Furthermore, perspectives of patients and surrogate decision makers were left unexplored in the included articles of this review, despite being potential end users of prognostic models. Incorporating these perspectives is crucial to improve prognostic risk communication and ensure these tools are patient centered. Future studies should also examine ethical concerns, including patient autonomy and reliance on technology, to ensure these models align with shared decision-making principles.

Due to limited reporting on participant characteristics, such as age, gender, prior experience, or voluntariness of use, no insights could be drawn on how these moderating factors from the UTAUT framework influence the adoption of prognostic models. Future studies should collect sociodemographic data to clarify their impact on model adoption and guide tailored implementation strategies. Larger, well-defined clinician samples are needed to clarify how these tools are adopted in practice. In addition, examining how variations in clinician demographics, such as professional background, experience level, and technological familiarity, affect the implementation of models could offer valuable insights into implementation challenges.

Beyond individual clinician characteristics, broader cultural and systemic factors are also likely to influence the implementation of prognostic models. Future research should examine how cultural and systemic factors influence technology adoption in health care, particularly how organizational culture, health care policies, and regional practices shape the adoption of new technologies.

To enhance the generalizability of findings, future research should incorporate larger datasets that allow for meaningful subgroup analysis. In addition, expanding the scope by incorporating gray literature, with rigorous appraisal methods to ensure credibility, could provide insights into real-world implementation barriers.

Future research should develop and evaluate implementation strategies that promote collaboration among researchers, clinicians, patients, and surrogate decision makers to bridge the gap between model development and real-world application. Emphasizing iterative processes, end-user feedback, and tailored clinician training will aid integration into clinical care. In addition, iterative co-design and co-creation methods, such as feedback sessions and idea-sharing platforms, are key to overcoming adoption barriers and ensuring successful implementation.

## Conclusion

This systematic review provided an extensive overview of facilitators and barriers as perceived by clinicians on the usage of prognostic models for clinical decision making in acute neurologic care. Commonly mentioned facilitators included improvements in communication with patients and surrogate decision makers, reassurance of clinical judgement, and improvements in patient outcomes. Commonly mentioned barriers included reluctance in use during risk communication, mistrust in data, and perceived reduction of autonomy. Understanding these facilitators and barriers prior to model development is crucial, as it can guide the design and implementation of prognostic models. To facilitate implementation in clinical practice, it is essential to foster collaborations between researchers from various fields, clinicians, patients, and their surrogate decision makers to bridge the gap between model development and clinical implementation in practice.

## Supplemental Material

sj-docx-1-mdm-10.1177_0272989X251343027 – Supplemental material for Facilitators and Barriers of the Use of Prognostic Models for Clinical Decision Making in Acute Neurologic Care: A Systematic Review Supplemental material, sj-docx-1-mdm-10.1177_0272989X251343027 for Facilitators and Barriers of the Use of Prognostic Models for Clinical Decision Making in Acute Neurologic Care: A Systematic Review by Ellen X. Y. Hu, Evelien S. van Hoorn, Isabel R. A. Retel Helmrich, Susanne Muehlschlegel, Judith A. C. Rietjens and Hester F. Lingsma in Medical Decision Making

sj-docx-2-mdm-10.1177_0272989X251343027 – Supplemental material for Facilitators and Barriers of the Use of Prognostic Models for Clinical Decision Making in Acute Neurologic Care: A Systematic Review Supplemental material, sj-docx-2-mdm-10.1177_0272989X251343027 for Facilitators and Barriers of the Use of Prognostic Models for Clinical Decision Making in Acute Neurologic Care: A Systematic Review by Ellen X. Y. Hu, Evelien S. van Hoorn, Isabel R. A. Retel Helmrich, Susanne Muehlschlegel, Judith A. C. Rietjens and Hester F. Lingsma in Medical Decision Making
