# Supplementary material for: Facilitators and Barriers of the Use of Prognostic Models for Clinical Decision Making in Acute Neurologic Care: A Systematic Review
Source: Med Decis Making. 2025 Jun 29;45(6):753–70. doi: 10.1177/0272989X251343027 (PMC12260205; doi:10.1177/0272989X251343027)
Supplement: sj-docx-1-mdm-10.1177_0272989X251343027 – Supplemental material for Facilitators and Barriers of the Use of Prognostic Models for Clinical Decision Making in Acute Neurologic Care: A Systematic Review [file sj-docx-1-mdm-10.1177_0272989X251343027.docx]

**Appendix 1.** Search strategy

**Embase.com**

('traumatic brain injury'/exp OR 'cerebrovascular accident'/exp OR 'brain hemorrhage'/exp OR 'brain infarction'/exp OR 'anoxic brain injury'/de OR (TBI OR ((trauma* OR posttrauma*) NEAR/3 (brain* OR cerebr*) NEAR/3 (injur* OR lesion*)) OR ((trauma* OR posttrauma*) NEAR/3 (encephalopath* OR brain* OR cerebr*)) OR CVA OR ((cerebr* OR brain-vascular*) NEAR/3 (accident* OR insult* OR lesion* OR failure*)) OR stroke* OR ((brain* OR cerebr* OR cerebell* OR corpus-callosum* OR intracranial* OR intraventricular* OR periventricular* OR intracerebral* OR posterior-fossa* OR subarachnoid* OR arachnoid*) NEAR/3 (hemorrhage* OR haemorrhage OR microhaemorrhage* OR microhemorrhage* OR bleed* OR microbleed* OR hematoma* OR haematoma* OR infarct*)) OR ((anox*) NEAR/4 (brain*) NEAR/4 (injur*))):ab,ti,kw) **AND** ((('prognosis'/de OR 'adverse outcome'/de OR 'prediction'/de OR 'decision making'/de) AND ('statistical model'/exp)) OR 'clinical decision support system'/de OR 'decision support system'/de OR (((prognos* OR decision* OR predict* OR adverse-outcome*) NEAR/3 (model*)) OR CRASH OR corticosteroid-randomi*-after-significant-head-injur* OR international-mission-for-prognosis-and-clinical-trial* OR risk-function* OR decision-support-system*):ab,ti,kw) **AND** ('implementation science'/exp OR 'evaluation study'/exp OR 'clinical decision making'/de OR 'health personnel attitude'/de OR 'physician'/de OR (implement* OR facilitator* OR barrier* OR ((evaluat*) NEAR/6 (stud*)) OR ((physician* OR clinic*) NEAR/3 (percept* OR perceive*)) OR clinical-decision*):ab,ti,kw) NOT [conference abstract]/lim

**Medline (Ovid)**

(exp Brain Injuries, Traumatic/ OR exp Stroke/ OR exp Intracranial Hemorrhages/ OR exp Hypoxia, Brain/ OR (TBI OR ((trauma* OR posttrauma*) ADJ3 (brain* OR cerebr*) ADJ3 (injur* OR lesion*)) OR ((trauma* OR posttrauma*) ADJ3 (encephalopath* OR brain* OR cerebr*)) OR CVA OR ((cerebr* OR brain-vascular*) ADJ3 (accident* OR insult* OR lesion* OR failure*)) OR stroke* OR ((brain* OR cerebr* OR cerebell* OR corpus-callosum* OR intracranial* OR intraventricular* OR periventricular* OR intracerebral* OR posterior-fossa* OR subarachnoid* OR arachnoid*) ADJ3 (hemorrhage* OR haemorrhage OR microhaemorrhage* OR microhemorrhage* OR bleed* OR microbleed* OR hematoma* OR haematoma* OR infarct*)) OR ((anox*) ADJ4 (brain*) ADJ4 (injur*))).ab,ti,kw.) **AND** (Nomograms/ OR ((exp Prognosis/ OR Decision Making/) AND (Models, Statistical/)) OR Decision Support Systems, Clinical/ OR (((prognos* OR decision* OR predict* OR adverse-outcome*) ADJ3 (model*)) OR CRASH OR corticosteroid-randomi*-after-significant-head-injur* OR international-mission-for-prognosis-and-clinical-trial* OR risk-function* OR decision-support-system*).ab,ti,kw.) **AND** (Implementation Science/ OR Evaluation Study.pt. OR Clinical Decision-Making/ OR Attitude of Health Personnel/ OR Physicians/ OR (implement* OR facilitator* OR barrier* OR ((evaluat*) ADJ6 (stud*)) OR ((physician* OR clinic*) ADJ3 (percept* OR perceive*)) OR clinical-decision*).ab,ti,kw.) NOT (news OR congres* OR abstract* OR book* OR chapter* OR dissertation abstract*).pt.

**Web of Science**

TS=(((TBI OR ((trauma* OR posttrauma*) NEAR/2 (brain* OR cerebr*) NEAR/2 (injur* OR lesion*)) OR ((trauma* OR posttrauma*) NEAR/2 (encephalopath* OR brain* OR cerebr*)) OR CVA OR ((cerebr* OR brain-vascular*) NEAR/2 (accident* OR insult* OR lesion* OR failure*)) OR stroke* OR ((brain* OR cerebr* OR cerebell* OR corpus-callosum* OR intracranial* OR intraventricular* OR periventricular* OR intracerebral* OR posterior-fossa* OR subarachnoid* OR arachnoid*) NEAR/2 (hemorrhage* OR haemorrhage OR microhaemorrhage* OR microhemorrhage* OR bleed* OR microbleed* OR hematoma* OR haematoma* OR infarct*)) OR ((anox*) NEAR/4 (brain*) NEAR/4 (injur*)))) **AND** ((((prognos* OR decision* OR predict* OR adverse-outcome*) NEAR/2 (model*)) OR CRASH OR corticosteroid-randomi*-after-significant-head-injur* OR international-mission-for-prognosis-and-clinical-trial* OR risk-function* OR decision-support-system*)) **AND** ((implement* OR facilitator* OR barrier* OR ((evaluat*) NEAR/5 (stud*)) OR ((physician* OR clinic*) NEAR/2 (percept* OR perceive*)) OR clinical-decision*))) AND DT=(Article OR Review OR Early Access OR Letter)

**Cochrane Central**

((TBI OR ((trauma* OR posttrauma*) NEAR/3 (brain* OR cerebr*) NEAR/3 (injur* OR lesion*)) OR ((trauma* OR posttrauma*) NEAR/3 (encephalopath* OR brain* OR cerebr*)) OR CVA OR ((cerebr* OR brain-vascular*) NEAR/3 (accident* OR insult* OR lesion* OR failure*)) OR stroke* OR ((brain* OR cerebr* OR cerebell* OR corpus-callosum* OR intracranial* OR intraventricular* OR periventricular* OR intracerebral* OR posterior-fossa* OR subarachnoid* OR arachnoid*) NEAR/3 (hemorrhage* OR haemorrhage OR microhaemorrhage* OR microhemorrhage* OR bleed* OR microbleed* OR hematoma* OR haematoma* OR infarct*)) OR ((anox*) NEAR/4 (brain*) NEAR/4 (injur*))):ab,ti,kw) **AND** ((((prognos* OR decision* OR predict* OR adverse-outcome*) NEAR/6 (model*)) OR CRASH OR (corticosteroid-randomi* NEXT after-significant-head-injur*) OR "international mission for prognosis and clinical trial*" OR risk-function* OR decision-support-system*):ab,ti,kw) **AND** ((implement* OR facilitator* OR barrier* OR ((evaluat*) NEAR/3 (stud*)) OR ((physician* OR clinic*) NEAR/3 (percept* OR perceive*)) OR clinical-decision*):ab,ti,kw) NOT "conference abstract":pt

Additional search

**Embase.com**

('traumatic brain injury'/mj/exp OR 'cerebrovascular accident'/mj/exp OR 'brain hemorrhage'/mj/exp OR 'brain infarction'/mj/exp OR 'anoxic brain injury'/mj/de OR (TBI OR ((trauma* OR posttrauma*) NEAR/3 (brain* OR cerebr*) NEAR/3 (injur* OR lesion*)) OR ((trauma* OR posttrauma*) NEAR/3 (encephalopath* OR brain* OR cerebr*)) OR CVA OR ((cerebr* OR brain-vascular*) NEAR/3 (accident* OR insult* OR lesion* OR failure*)) OR stroke* OR ((brain* OR cerebr* OR cerebell* OR corpus-callosum* OR intracranial* OR intraventricular* OR periventricular* OR intracerebral* OR posterior-fossa* OR subarachnoid* OR arachnoid*) NEAR/3 (hemorrhage* OR haemorrhage OR microhaemorrhage* OR microhemorrhage* OR bleed* OR microbleed* OR hematoma* OR haematoma* OR infarct*)) OR ((anox*) NEAR/4 (brain*) NEAR/4 (injur*))):ti) **AND** ((('prognosis'/mj/de OR 'adverse outcome'/mj/de OR 'prediction'/mj/de OR 'decision making'/mj/de) AND ('statistical model'/mj/exp)) OR 'clinical decision support system'/mj/de OR 'decision support system'/mj/de OR (((prognos* OR decision* OR predict* OR adverse-outcome*) AND (model*)) OR CRASH OR corticosteroid-randomi*-after-significant-head-injur* OR international-mission-for-prognosis-and-clinical-trial* OR risk-function* OR decision-support-system*):ti) NOT [conference abstract]/lim

**Medline (Ovid)**

(exp * Brain Injuries, Traumatic/ OR exp * Stroke/ OR exp * Intracranial Hemorrhages/ OR exp * Hypoxia, Brain/ OR (TBI OR ((trauma* OR posttrauma*) ADJ3 (brain* OR cerebr*) ADJ3 (injur* OR lesion*)) OR ((trauma* OR posttrauma*) ADJ3 (encephalopath* OR brain* OR cerebr*)) OR CVA OR ((cerebr* OR brain-vascular*) ADJ3 (accident* OR insult* OR lesion* OR failure*)) OR stroke* OR ((brain* OR cerebr* OR cerebell* OR corpus-callosum* OR intracranial* OR intraventricular* OR periventricular* OR intracerebral* OR posterior-fossa* OR subarachnoid* OR arachnoid*) ADJ3 (hemorrhage* OR haemorrhage OR microhaemorrhage* OR microhemorrhage* OR bleed* OR microbleed* OR hematoma* OR haematoma* OR infarct*)) OR ((anox*) ADJ4 (brain*) ADJ4 (injur*))).ti.) **AND** (* Nomograms/ OR ((exp * Prognosis/ OR * Decision Making/) AND (* Models, Statistical/)) OR * Decision Support Systems, Clinical/ OR (((prognos* OR decision* OR predict* OR adverse-outcome*) ADJ6 (model*)) OR CRASH OR corticosteroid-randomi*-after-significant-head-injur* OR international-mission-for-prognosis-and-clinical-trial* OR risk-function* OR decision-support-system*).ti.)

**Appendix 2a.** Predefined eligibility criteria for the title and abstract screening process

**#1 Is the full text of the article available?**
YES Proceed to #2
No Code EXCLUDE1 and STOP

**#2 Is the full text of the article in English?**
YES Proceed to #3
NO Code EXCLUDE 2 and STOP

**#3 Is the article a conference abstract?**
YES CODE EXCLUDE3 and STOP
NO Proceed to #4

**#4 Does the article have a healthcare context?**
YES Proceed to # 5
NO CODE EXCLUDE4 and STOP

**#5 Is the article an empirical study?**
YES Proceed to #6
NO Code EXCLUDE5 and STOP

**#6 Does the article mention TBI or other similar serious neurological diseases?**
YES Proceed to #7
NO Code EXCLUDE6 and STOP

**#7 Does the article mention usage of implementation of prognostic models in clinical care of acute neurological diseases?**
YES Proceed to #8
NO CODE EXCLUDE7 and STOP

**#8 Does the article provide an evaluation about the usage and implementation of prognostic models from clinicians’ perspective?**
YES Include the article
NO Code EXCLUDE8 and STOP

**Appendix 2b.** Predefined eligibility criteria for the full text screening process

1. No access to full article
2. The article is a non-English publication
3. The article is a non-empirical study
4. Article has no healthcare context
5. Article does not mention TBI or other serious acute neurological diseases as main outcome of interest
6. Article does not mention the implementation of prognostic modelling in clinical care of acute neurological diseases
7. Article does not provide a qualitative evaluation about the usage or implementation of prognostic models from clinician's perspective

*(shortened for Covidence)*

1. *No access to full article*
2. *The article is a non-Englisch publication*
3. *Article is a non-empirical study*
4. *Article has no healthcare context*
5. *TBI or other serious neurological diseases not as main outcome*
6. *Implementation of prognostic models not mentioned*
7. *No qualitative evaluation provided*

**Appendix 3.**  Summary of included studies and prognostic models under the TiDieR checklist items

| TiDieR checklist item | Brief name | Why (Rationale, theory, goal) | What (materials) | What (procedures) | Who provided | How | Where | When and How Much | Tailoring | Modifications | How well (planned) | How well (actual) |
| --- | --- | --- | --- | --- | --- | --- | --- | --- | --- | --- | --- | --- |
| Study  (First author, year),  *[related study]* |  |  |  |  |  |  |  |  |  |  |  |  |
| Amann, 2023^a 20^ | ? | ? | ? | ? | ? | ? | ? | ? | ? | ? | ? | ? |
| Elahi, 2020 ^30^ | A traumatic brain injury decision support tool (p.1) | ? | TBI risk calculator app  (p.2,3) | ? | ? | Face-to-face, individually in controlled setting  (p.3) | Two referral hospitals (p.2) | ? | ? | ? | ? | ? |
| *[Rocha, 2020]* ^57^ | ? | To optimize resources and predict outcomes using accessible, non-resource-intensive data (p.1,2) | ? | Development of a TBI prognostic model using machine learning (p.2,3,5,7) | Healthcare providers and decision-makers (p.1,2) | ? | ? | ? | ? | ? | ? | ? |
| Flynn, 2015 ^31^ | Computerized decision aid for thrombolysis in acute stroke care  (p.1) | To enhance shared decision making with individualized risk predictions (p.2) | Workshops, usability testing with paper and Ipad tools  (p.1) | Development of decision model, workshops, tests with stakeholder feedback (p.3,4) | Stroke physicians and stroke nurses (p.6) | Face-to-face between stroke clinicians and with patients/relatives (p.6) | Acute stroke units and clinical settings  (p.2) | Developed, tested and refined over multiple phases  (p.3,7) | Supports personalized decisions with real-time updates (p.5,12) | Modified post-alpha to enhance usability, clarity, acceptability (p.4,8) | ? | ? |
| Ghandour, 2020  ^32^ | Head CT Choice decision aid &  Concussion or Brain Bleed decision aid  (p.3) | ? | ? | ? | ? | ? | ? | ? | ? | ? | ? | ? |
| *[Hess, 2014]* ^58^ | ? | To evaluate the Head CT Choice aid’s impact on shared decision-making and parent empowerment  (p.1,7,8) | Paper decision aid guide  (p.5) | Training through lectures and video, assessment through recordings  (p.8,9) | Faculty physicians, fellows, nurses, physician assistants (p.6) | Group rounds and training (face-to-face), video demonstrations (online) (p.5) | Emergency departments, with video recording and telephone follow-up  (p.2,3) | ? | Personalized per patient based on their ciTBI risk  (p.5) | ? | ? | ? |
| *[Melnick,2015]* ^59^ | ? | To optimize CT use for minor head injuries by enhancing shared decision-making and communication (p.6,8,13) | Tablet with educational visuals (p.8,9) | ? | Emergency physicians, nurses, emergency physicians, nurses  (p.13) | Face-to-face, using a shared tablet  (p.6,13) | Emergency department, using a tablet  (p.6,7) | ? | Tailored to needs and circumstances of the patient  (p.3,10) | Modified during development to improve usability and patient-provider communication  (p.9) | ? | ? |
| Greenberg, 2021 ^21^ | Electronic CDS for children with minor head trauma and intercranial injuries  (p.1) | To guide admissions, standardize practices, prevent unnecessary transfer  (p.4,8) | Wireframe prototype  (p.3) | ? | Physicians from various specialties and other stakeholders  (p.4,6) | ? | Various locations, including academic hospitals and community medical center  (p.2,3) | ? | ? | ? | ? | ? |
| *[Greenberg, 2017]* ^60^ | ? | ? | ? | Development through regression, validation, calibration, data imputation  (p.3) | ? | ? | ? | ? | ? | ? | ? | ? |
| He, 2023 ^34^ | AI thrombolytic assistant, embedded within an AI-CDSS  (p.11) | To meet physicians’ needs, seamless integration in workflow  (p.8) | Prototype evaluation materials for feedback  (p.18) | Needs assessment, prototype design, user instruction, evaluation, iterations  (p.11,13) | Physicians  (p.11) | Online in group setting for clinicians. Unknown for patients.  (p.18) | County-level hospitals, requiring internet access and stroke-related facilities  (p.12) | Multiple iterative prototype evaluations, seven-month period  (p.11) | Personalization through interviews, iterative development based on physician’s feedback  (p.12) | ? | ? | ? |
| Kiatchai, 2017 ^26^ | Real-time CDS system for anesthetic management of pediatric TBI  (p.2) | To improve pediatric TBI care with real-time guideline reminders  (p.2) | CDS system, integrated in information management systems  (p.5) | The system went through several phases. Support activities include education, feedback collection  (p.3,4,5) | Anesthesiologists  (p.5) | Face-to-face meetings, emails, presentations, and one-on-one discussions.  (p.5) | Piloted in operating rooms  (p.4) | ? | Tailored management during surgery by using patient specific data  (p.3) | Sampling frequency, safeguards, updates, and decision rules were adjusted  (p.2,5,6) | ? | ? |
| Liberman, 2022^a 22^ | ? | ? | ? | ? | ? | ? | ? | ? | ? | ? | ? | ? |

| Masterson Creber, 2018 ^29^ | Clinical decision support tool for pediatric head trauma  (p.2) | ? | Clinical decision support tool  (p.3) | Tool integration, change process investment, user training  (p.2,5) | ? | ? | Community ED and academic hospitals, requiring tool integration, process investment, training  (p.2,3) | ? | ? | ? | ? | ? |
| --- | --- | --- | --- | --- | --- | --- | --- | --- | --- | --- | --- | --- |
| *[Kupperman, 2009]^b^* ^61^ | ? | To identify low-risk children for CT, reducing radiation risk, aiding decision making  (p.1,4,9,10) | ? | ? | Emergency department clinicians  (p.3) | ? | Emergency departments  (p.2) | ? | ? | ? | ? | ? |
| *[Dayan, 2017]* ^62^ | ? | To evaluate PECARN rule’s effectiveness in predicting ciTBI and guiding CT use in children  (p2.5) | ? | ? | Emergency physicians (p.6) | Face-to-face, individually (p.3) | Emergency departments (p.2) | ? | ? | ? | ? | ? |
| Moskowitz, 2018 ^23^ | Decision aid for ciTBI patients (IMPACT-model) (p.1) | To provide numeric risk etimates, reduce prognostic variability  (p.1) | ? | ? | ? | ? | ? | ? | ? | ? | ? | ? |
| *[Steyerberg,2008]* ^10^ | ? | ? | IMPACT model includes patient characteristics, CCS motor score, CT features and biomechamical variables  (p.2) | Developed using key predictors, logistic regression, and AUC performance assessment  (p.2,3) | ? | ? | ? | ? | ? | ? | ? | ? |
| O’Leary, 2023 ^25^ | TBI predictive modeling tool/ TBI tool  (p.1,3) | To enhance physicians’ diagnostic ability, aiding effective treatment decisions  (p.1) | Web-based application of the tool  (p.2,6) | Co-design with healthcare staff (p.3,4) | Physicians/ healthcare providers  (p.1,2) | Individually, virtual communication between physicians/ hospitals  (p.2,7,8) | Medical center, university hospital  (p.3) | ? | Adaptations based on co-design sessions, tailored to fit in current workflow | ? | ? | ? |
| Ranta, 2013 ^27^ | TIA/ Stroke electronic decision support tool (p.1) | To aid in diagnosis, triaging, and treatment of patients with TIAs/ stroke  (p.1) | Web-based electronic decision support tool (p.1) | Pilot implementation, tool usage training in groups  (p.1,2) | General practitioners (p.3) | Face-to-face, delivery in groups  (p.2) | Primary practices (p.1,2) | One training session, pilot for eight weeks (p.2) | ? | Override option (p.3) | ? | ? |
| Sheehan, 2013 ^24^ | Clinical decision support system intervention (p.1,2) | To improve evaluation, reduce errors and enhance quality by integrating the PECARN (p.1,2) | ? | Sociotechnical analysis, workflow observations (p.1,2) | Clinicians at the ED (p.3) | Face-to-face, delivery in groups (p.2,3) | Emergency departments (p.2) | Pilot for four months (p.2) | ? | ? | ? | ? |
| *[Kupperman, 2009] ^b^* ^61^ |  |  |  |  |  |  |  |  |  |  |  |  |
| Yadav, 2015 ^33^ | Electronic clinical decision support tool  (p.2) | To provide real-time guidance to reduce unnecessary head CTs in children  (p.7) | Prototype electronic decision support  (p.2) | Development through prototype design, task analysis and heuristic evaluation  (p.2,3) | Pediatric emergency physicians, trauma resuscitation teams  (p.1) | Online prototype, delivery in groups  (p.2) | Medical center  (p.2) | Delivery over multiple sessions (p.3) | Personalization through human factor approach  (p.5,7) | Modifications include rephrasing questions, positive phrasing, including intermediate feedback  (p.4,5) | ? | ? |
| *[Kupperman, 2009] ^b^* ^61^ |  |  |  |  |  |  |  |  |  |  |  |  |
| Zakhari, 2016 ^28^ | Canadian CT head rule  (p.2) | To reduce CT exposure to patients (p.1,3) | Physical and informational materials, educational sessions, knowledge assessments  (p.4,5) | ? | Emergency physicians (p.4) | Face-to-face, individual  (p.4) | ED of community and university hospitals  (p.2) | 6 weeks, weekly individual meetings and education sessions  (p.4) | ? | ? | ? | ? |
| *[Stiell, 2001]* ^63^ | ? | ? | Standardized patient assessment sheets  (p.2) | Standardized data collection, interobserver agreement checks, 14-day follow-up (p.2,3) | ? | ? | ? | ? | ? | ? | ? | ? |

? = Not reported/ not sufficiently reported
^1^ The article by Amann and Liberman examines the use of prognostic models in general, focusing on end-users’ perceptions rather than model development or validation. No additional articles on a specific model were found.
^2^ Same study (Kupperman, 2009)

Abbreviations: TBI, Traumatic Brain Injury; CT, computed tomography; ciTBI, critically ill Traumatic Brain Injury; CDS, Clinical Decision Support; AI, Artificial Intelligence; AI-CDSS, Artificial Intelligence Clinical Decision Support System; ED, Emergency Department; PERCARN, the Pediatric Emergency Care Applied Research Network; IMPACT, International Mission for Prognosis And Clinical Trial; CCS, Clinical Classification System; AUC, Area Under the Curve; TIA, Transient Ischemic Attack

**Appendix 4a.** Quality assessment scores for the included studies – qualitative studies

| Author (Reference) | Objective | Study design | Context | Theoretical framework | Sampling strategy | Data collection | Data analysis | Verification procedures | Conclusion | Reflexivity | **Total sum** | **Summary score** | **Quality** |
| --- | --- | --- | --- | --- | --- | --- | --- | --- | --- | --- | --- | --- | --- |
| Amann^20^ | 2 | 2 | 2 | 2 | 2 | 2 | 2 | 2 | 2 | 1 | 19 | 0.95 | Strong |
| Elahi^30^ | 2 | 2 | 2 | 2 | 1 | 1 | 1 | 0 | 2 | 0 | 13 | 0.8^1^ | Good |
| Flynn^31^ | 2 | 2 | 0 | 1 | 0 | 1 | 1 | 2 | 2 | 1 | 12 | 0.68^1^ | Adequate |
| Ghandour^32^ | 2 | 2 | 2 | 0 | 2 | 2 | 1 | 2 | 2 | 1 | 16 | 0.87^1^ | Strong |
| Greenberg^60^ | 2 | 2 | 2 | 2 | 2 | 2 | 2 | 2 | 2 | 0 | 18 | 0.9 | Strong |
| He^34^ | 2 | 2 | 2 | 1 | 2 | 2 | 2 | 2 | 2 | 0 | 17 | 0.77^1^ | Good |
| Liberman^22^ | 2 | 2 | 2 | 2 | 2 | 2 | 2 | 2 | 2 | 1 | 19 | 0.95 | Strong |
| Masterson Creber^29^ | 2 | 2 | 2 | 2 | 1 | 2 | 2 | 2 | 2 | 1 | 18 | 0.95^1^ | Strong |
| Moskowitz^23^ | 2 | 2 | 2 | 1 | 2 | 2 | 2 | 2 | 2 | 0 | 17 | 0.85 | Strong |
| O’Leary^25^ | 2 | 2 | 2 | 2 | 2 | 2 | 2 | 2 | 2 | 1 | 19 | 0.95 | Strong |
| Sheehan^24^ | 2 | 2 | 2 | 2 | 2 | 2 | 2 | 2 | 2 | 0 | 18 | 0.9 | Strong |
| Yadav^33^ | 2 | 2 | 2 | 2 | 1 | 2 | 1 | 2 | 1 | 1 | 16 | 0.68^1^ | Adequate |

^1^ Mixed-method study: the summary score consists of the sum of both qualitative as quantitative assessment divided by two.
N/A: not applicable

**Appendix 4b.** Quality assessment scores for the included studies – quantitative studies

| Author (Reference) | Objective | Study design | Study selection | Study characteristics | Random allocation | Blinding of investigator | Blinding of subjects | Outcome measures | Sample size | Analytic methods | Estimate of variance | Control for confounding | Result | Conclusion | **Total sum** | **Summary score** | **Quality** |
| --- | --- | --- | --- | --- | --- | --- | --- | --- | --- | --- | --- | --- | --- | --- | --- | --- | --- |
| Elahi^30^ | 2 | 2 | 2 | 2 | N/A | N/A | N/A | 1 | 2 | N/A | N/A | N/A | 2 | 2 | 15 | 0.8^1^ | Good |
| Flynn^31^ | 2 | 2 | 1 | 0 | N/A | N/A | N/A | 2 | 1 | N/A | N/A | N/A | 2 | 2 | 12 | 0.68^1^ | Adequate |
| Ghandour^32^ | 2 | 2 | 2 | 2 | N/A | N/A | N/A | 1 | N/A | N/A | N/A | N/A | 2 | 2 | 13 | 0.87^1^ | Strong |
| He^34^ | 2 | 1 | 2 | 2 | N/A | N/A | N/A | 2 | N/A | N/A | N/A | N/A | 1 | 2 | 12 | 0.77^1^ | Good |
| Kiatchai^26^ | 2 | 1 | 1 | 0 | N/A | N/A | N/A | 2 | N/A | N/A | N/A | N/A | 2 | 2 | 10 | 0.71 | Good |
| Masterson Creber^29^ | 2 | 2 | 2 | 2 | N/A | N/A | N/A | 2 | N/A | NA | N/A | N/A | 2 | 2 | 14 | 0.95^1^ | Strong |
| Ranta^27^ | 2 | 1 | 1 | 1 | N/A | N/A | N/A | 1 | N/A | 0 | N/A | N/A | 1 | 1 | 8 | 0.5 | Low |
| Yadav^33^ | 2 | 2 | 1 | 0 | N/A | N/A | N/A | 0 | 1 | N/A | N/A | N/A | 1 | 2 | 9 | 0.68^1^ | Good |
| Zakhari^28^ | 2 | 2 | 2 | 1 | N/A | N/A | N/A | 2 | N/A | 2 | N/A | N/A | 2 | 2 | 15 | 0.94 | Strong |

^1^ Mixed-method study: the summary score consists of the sum of both qualitative as quantitative assessment divided by two.
N/A: not applicable

**References**

57. Rocha TAH, Elahi C, da Silva NC, et al. A traumatic brain injury prognostic model to support in-hospital triage in a low-income country: a machine learning–based approach. J Neurosurg. 2020;132:1961–9.

58. Hess EP, Wyatt KD, Kharbanda AB, et al. Effectiveness of the head CT choice decision aid in parents of children with minor head trauma: study protocol for a multicenter randomized trial. Trials. 2014;15:253.

59. Melnick ER, Lopez K, Hess EP, et al. Back to the bedside: developing a bedside aid for concussion and brain injury decisions in the emergency department. EGEMS. 2015;3:1136.

60. Greenberg JK, Yan Y, Carpenter CR, et al. Development and internal validation of a clinical risk score for treating children with mild head trauma and intracranial injury. JAMA Pediatr. 2017;171:342.

61. Kuppermann N, Holmes JF, Dayan PS, et al. Identification of children at very low risk of clinically-important brain injuries after head trauma: a prospective cohort study. Lancet. 2009;374:1160–70.

62. Dayan PS, Ballard DW, Tham E, et al. Use of traumatic brain injury prediction rules with clinical decision support. Pediatrics. 2017;139:e20162709.

63. Stiell IG, Wells GA, Vandemheen K, et al. The Canadian CT Head Rule for patients with minor head injury. Lancet. 2001;357:1391–6.
