# Supplementary material for: Facilitators and Barriers of the Use of Prognostic Models for Clinical Decision Making in Acute Neurologic Care: A Systematic Review
Source: Med Decis Making. 2025 Jun 29;45(6):753–70. doi: 10.1177/0272989X251343027 (PMC12260205; doi:10.1177/0272989X251343027)
Supplement: sj-docx-2-mdm-10.1177_0272989X251343027 – Supplemental material for Facilitators and Barriers of the Use of Prognostic Models for Clinical Decision Making in Acute Neurologic Care: A Systematic Review [file sj-docx-2-mdm-10.1177_0272989X251343027.docx]

**Appendix 3.**  Summary of included studies and prognostic models under the TiDieR checklist items

| TiDieR checklist item | Brief name | Why (Rationale, theory, goal) | What (materials) | What (procedures) | Who provided | How | Where | When and How Much | Tailoring | Modifications | How well (planned) | How well (actual) |
| --- | --- | --- | --- | --- | --- | --- | --- | --- | --- | --- | --- | --- |
| Study  (First author, year),  *[related study]* |  |  |  |  |  |  |  |  |  |  |  |  |
| Amann, 2023^a 20^ | ? | ? | ? | ? | ? | ? | ? | ? | ? | ? | ? | ? |
| Elahi, 2020 ^30^ | A traumatic brain injury decision support tool (p.1) | ? | TBI risk calculator app  (p.2,3) | ? | ? | Face-to-face, individually in controlled setting  (p.3) | Two referral hospitals (p.2) | ? | ? | ? | ? | ? |
| *[Rocha, 2020]* ^57^ | ? | To optimize resources and predict outcomes using accessible, non-resource-intensive data (p.1,2) | ? | Development of a TBI prognostic model using machine learning (p.2,3,5,7) | Healthcare providers and decision-makers (p.1,2) | ? | ? | ? | ? | ? | ? | ? |
| Flynn, 2015 ^31^ | Computerized decision aid for thrombolysis in acute stroke care  (p.1) | To enhance shared decision making with individualized risk predictions (p.2) | Workshops, usability testing with paper and Ipad tools  (p.1) | Development of decision model, workshops, tests with stakeholder feedback (p.3,4) | Stroke physicians and stroke nurses (p.6) | Face-to-face between stroke clinicians and with patients/relatives (p.6) | Acute stroke units and clinical settings  (p.2) | Developed, tested and refined over multiple phases  (p.3,7) | Supports personalized decisions with real-time updates (p.5,12) | Modified post-alpha to enhance usability, clarity, acceptability (p.4,8) | ? | ? |
| Ghandour, 2020  ^32^ | Head CT Choice decision aid &  Concussion or Brain Bleed decision aid  (p.3) | ? | ? | ? | ? | ? | ? | ? | ? | ? | ? | ? |
| *[Hess, 2014]* ^58^ | ? | To evaluate the Head CT Choice aid’s impact on shared decision-making and parent empowerment  (p.1,7,8) | Paper decision aid guide  (p.5) | Training through lectures and video, assessment through recordings  (p.8,9) | Faculty physicians, fellows, nurses, physician assistants (p.6) | Group rounds and training (face-to-face), video demonstrations (online) (p.5) | Emergency departments, with video recording and telephone follow-up  (p.2,3) | ? | Personalized per patient based on their ciTBI risk  (p.5) | ? | ? | ? |
| *[Melnick,2015]* ^59^ | ? | To optimize CT use for minor head injuries by enhancing shared decision-making and communication (p.6,8,13) | Tablet with educational visuals (p.8,9) | ? | Emergency physicians, nurses, emergency physicians, nurses  (p.13) | Face-to-face, using a shared tablet  (p.6,13) | Emergency department, using a tablet  (p.6,7) | ? | Tailored to needs and circumstances of the patient  (p.3,10) | Modified during development to improve usability and patient-provider communication  (p.9) | ? | ? |
| Greenberg, 2021 ^21^ | Electronic CDS for children with minor head trauma and intercranial injuries  (p.1) | To guide admissions, standardize practices, prevent unnecessary transfer  (p.4,8) | Wireframe prototype  (p.3) | ? | Physicians from various specialties and other stakeholders  (p.4,6) | ? | Various locations, including academic hospitals and community medical center  (p.2,3) | ? | ? | ? | ? | ? |
| *[Greenberg, 2017]* ^60^ | ? | ? | ? | Development through regression, validation, calibration, data imputation  (p.3) | ? | ? | ? | ? | ? | ? | ? | ? |
| He, 2023 ^34^ | AI thrombolytic assistant, embedded within an AI-CDSS  (p.11) | To meet physicians’ needs, seamless integration in workflow  (p.8) | Prototype evaluation materials for feedback  (p.18) | Needs assessment, prototype design, user instruction, evaluation, iterations  (p.11,13) | Physicians  (p.11) | Online in group setting for clinicians. Unknown for patients.  (p.18) | County-level hospitals, requiring internet access and stroke-related facilities  (p.12) | Multiple iterative prototype evaluations, seven-month period  (p.11) | Personalization through interviews, iterative development based on physician’s feedback  (p.12) | ? | ? | ? |
| Kiatchai, 2017 ^26^ | Real-time CDS system for anesthetic management of pediatric TBI  (p.2) | To improve pediatric TBI care with real-time guideline reminders  (p.2) | CDS system, integrated in information management systems  (p.5) | The system went through several phases. Support activities include education, feedback collection  (p.3,4,5) | Anesthesiologists  (p.5) | Face-to-face meetings, emails, presentations, and one-on-one discussions.  (p.5) | Piloted in operating rooms  (p.4) | ? | Tailored management during surgery by using patient specific data  (p.3) | Sampling frequency, safeguards, updates, and decision rules were adjusted  (p.2,5,6) | ? | ? |
| Liberman, 2022^a 22^ | ? | ? | ? | ? | ? | ? | ? | ? | ? | ? | ? | ? |

| Masterson Creber, 2018 ^29^ | Clinical decision support tool for pediatric head trauma  (p.2) | ? | Clinical decision support tool  (p.3) | Tool integration, change process investment, user training  (p.2,5) | ? | ? | Community ED and academic hospitals, requiring tool integration, process investment, training  (p.2,3) | ? | ? | ? | ? | ? |
| --- | --- | --- | --- | --- | --- | --- | --- | --- | --- | --- | --- | --- |
| *[Kupperman, 2009]^b^* ^61^ | ? | To identify low-risk children for CT, reducing radiation risk, aiding decision making  (p.1,4,9,10) | ? | ? | Emergency department clinicians  (p.3) | ? | Emergency departments  (p.2) | ? | ? | ? | ? | ? |
| *[Dayan, 2017]* ^62^ | ? | To evaluate PECARN rule’s effectiveness in predicting ciTBI and guiding CT use in children  (p2.5) | ? | ? | Emergency physicians (p.6) | Face-to-face, individually (p.3) | Emergency departments (p.2) | ? | ? | ? | ? | ? |
| Moskowitz, 2018 ^23^ | Decision aid for ciTBI patients (IMPACT-model) (p.1) | To provide numeric risk etimates, reduce prognostic variability  (p.1) | ? | ? | ? | ? | ? | ? | ? | ? | ? | ? |
| *[Steyerberg,2008]* ^10^ | ? | ? | IMPACT model includes patient characteristics, CCS motor score, CT features and biomechamical variables  (p.2) | Developed using key predictors, logistic regression, and AUC performance assessment  (p.2,3) | ? | ? | ? | ? | ? | ? | ? | ? |
| O’Leary, 2023 ^25^ | TBI predictive modeling tool/ TBI tool  (p.1,3) | To enhance physicians’ diagnostic ability, aiding effective treatment decisions  (p.1) | Web-based application of the tool  (p.2,6) | Co-design with healthcare staff (p.3,4) | Physicians/ healthcare providers  (p.1,2) | Individually, virtual communication between physicians/ hospitals  (p.2,7,8) | Medical center, university hospital  (p.3) | ? | Adaptations based on co-design sessions, tailored to fit in current workflow | ? | ? | ? |
| Ranta, 2013 ^27^ | TIA/ Stroke electronic decision support tool (p.1) | To aid in diagnosis, triaging, and treatment of patients with TIAs/ stroke  (p.1) | Web-based electronic decision support tool (p.1) | Pilot implementation, tool usage training in groups  (p.1,2) | General practitioners (p.3) | Face-to-face, delivery in groups  (p.2) | Primary practices (p.1,2) | One training session, pilot for eight weeks (p.2) | ? | Override option (p.3) | ? | ? |
| Sheehan, 2013 ^24^ | Clinical decision support system intervention (p.1,2) | To improve evaluation, reduce errors and enhance quality by integrating the PECARN (p.1,2) | ? | Sociotechnical analysis, workflow observations (p.1,2) | Clinicians at the ED (p.3) | Face-to-face, delivery in groups (p.2,3) | Emergency departments (p.2) | Pilot for four months (p.2) | ? | ? | ? | ? |
| *[Kupperman, 2009] ^b^* ^61^ |  |  |  |  |  |  |  |  |  |  |  |  |
| Yadav, 2015 ^33^ | Electronic clinical decision support tool  (p.2) | To provide real-time guidance to reduce unnecessary head CTs in children  (p.7) | Prototype electronic decision support  (p.2) | Development through prototype design, task analysis and heuristic evaluation  (p.2,3) | Pediatric emergency physicians, trauma resuscitation teams  (p.1) | Online prototype, delivery in groups  (p.2) | Medical center  (p.2) | Delivery over multiple sessions (p.3) | Personalization through human factor approach  (p.5,7) | Modifications include rephrasing questions, positive phrasing, including intermediate feedback  (p.4,5) | ? | ? |
| *[Kupperman, 2009] ^b^* ^61^ |  |  |  |  |  |  |  |  |  |  |  |  |
| Zakhari, 2016 ^28^ | Canadian CT head rule  (p.2) | To reduce CT exposure to patients (p.1,3) | Physical and informational materials, educational sessions, knowledge assessments  (p.4,5) | ? | Emergency physicians (p.4) | Face-to-face, individual  (p.4) | ED of community and university hospitals  (p.2) | 6 weeks, weekly individual meetings and education sessions  (p.4) | ? | ? | ? | ? |
| *[Stiell, 2001]* ^63^ | ? | ? | Standardized patient assessment sheets  (p.2) | Standardized data collection, interobserver agreement checks, 14-day follow-up (p.2,3) | ? | ? | ? | ? | ? | ? | ? | ? |

? = Not reported/ not sufficiently reported
^1^ The article by Amann and Liberman examines the use of prognostic models in general, focusing on end-users’ perceptions rather than model development or validation. No additional articles on a specific model were found.
^2^ Same study (Kupperman, 2009)

Abbreviations: TBI, Traumatic Brain Injury; CT, computed tomography; ciTBI, critically ill Traumatic Brain Injury; CDS, Clinical Decision Support; AI, Artificial Intelligence; AI-CDSS, Artificial Intelligence Clinical Decision Support System; ED, Emergency Department; PERCARN, the Pediatric Emergency Care Applied Research Network; IMPACT, International Mission for Prognosis And Clinical Trial; CCS, Clinical Classification System; AUC, Area Under the Curve; TIA, Transient Ischemic Attack

**References**

57. Rocha TAH, Elahi C, Silva NC da, et al. A traumatic brain injury prognostic model to support in-hospital triage in a low-income country: a machine learning–based approach. *J Neurosurg JNS* 2020; 132: 1961–1969.

58. Hess EP, Wyatt KD, Kharbanda AB, et al. Effectiveness of the head CT choice decision aid in parents of children with minor head trauma: study protocol for a multicenter randomized trial. *Trials* 2014; 15: 253.

59. Melnick ER, Lopez K, Hess EP, et al. Back to the Bedside: Developing a Bedside Aid for Concussion and Brain Injury Decisions in the Emergency Department. *EGEMs Gener Evid Methods Improve Patient Outcomes* 2015; 3: 6.

60. Greenberg JK, Yan Y, Carpenter CR, et al. Development and Internal Validation of a Clinical Risk Score for Treating Children With Mild Head Trauma and Intracranial Injury. *JAMA Pediatr* 2017; 171: 342.

61. Kuppermann N, Holmes JF, Dayan PS, et al. Identification of children at very low risk of clinically-important brain injuries after head trauma: a prospective cohort study. *The Lancet* 2009; 374: 1160–1170.

62. Dayan PS, Ballard DW, Tham E, et al. Use of Traumatic Brain Injury Prediction Rules With Clinical Decision Support. *Pediatrics* 20

63. Stiell IG, Wells GA, Vandemheen K, et al. The Canadian CT Head Rule for patients with minor head injury. *The Lancet* 2001; 357: 1391–1396.
